# Awareness, knowledge, attitude, and skills of telemedicine among mental healthcare providers

**DOI:** 10.1186/s43045-022-00272-3

**Published:** 2023-01-11

**Authors:** Amira Ahmed Fouad, Mahmood Ahmed Osman, Yasmin Mohamed Mohamed Abdelmonaem, Nashwa Ahmed Hussein Abdel Karim

**Affiliations:** 1 Professor of Psychiatry, Faculty of Medicine, Zagazig University, Zagazig, Egypt; 2 Bachelor of Nursing, Faculty of Nursing, Zagazig University, Zagazig, Egypt; 3 Lecturer of Psychiatric and Mental Health Nursing Department, Faculty of Nursing, Zagazig University, Zagazig, Egypt

**Keywords:** Awareness, Knowledge, Attitude, Skills, Telemedicine, Mental healthcare providers

## Abstract

**Background:**

Telemedicine plays a vital role in patient-centered healthcare delivery in the diagnosis, management, and development of future treatment plans for chronic diseases.

**Results:**

This study revealed that attitude toward telemedicine was high among the studied mental healthcare providers, while it was average among the other studied variables (awareness, knowledge, and skills). Psychiatrists had a high percentage in the studied variables (awareness, attitude, knowledge, and skills). There were statistically significant differences in the dimensions of the telemedicine questionnaire according to age, profession, and academic degree. There was a statistically significant relation between the levels of awareness, knowledge, and attitude toward telemedicine.

**Conclusions:**

This study concluded that the studied mental healthcare providers had high attitudes while the other studied variables (awareness, knowledge, and skills) were average. Psychiatrists had a high percentage in the studied variables (awareness, attitude, knowledge, and skills), followed by psychologists, while nurses had the lowest level in all studied variables.

## Background

Today, telemedicine is a topic of great interest, but it is still unknown in the developing world. Global obstacles, such as the accessibility of high-quality and cost-effective services, can be potentially overcome by the right application of this technology in the healthcare system. For telemedicine to reach its full potency, it is necessary to define the attitude of patients and healthcare professionals toward this technology (Ashfaq et al., 2020).

The World Health Organization has defined telemedicine as the provision of health services by all health professionals who use technologies of information and communication to exchange correct information for the diagnosis, treatment, and prevention of diseases; research and evaluation; and continuous training of health professionals, all this to improve the health of individuals and communities [17].

Telemedicine helps healthcare providers monitor chronic diseases by enabling them to record physiological signs that are not observable during clinical visits [3]. Improving the health of individuals and communities is the main purpose of telemedicine through the exchange of useful information for various purposes, such as disease prevention, diagnosis, monitoring, provision of treatment, education of healthcare providers, and research (Gohari & Bahaadinbeigy, 2021).

Mental healthcare professional is defined as a person who is professionally qualified to provide counseling interventions designed to facilitate individual achievement of human development goals and to treat mental, emotional, or behavioral disorders as well as related distresses that interfere with mental health and development. Most have at least a master’s degree or more advanced education, training, and credentials (Clinic Staff, 2017).

Awareness was defined as the state or capacity to observe or be alert to circumstances or sensory patterns. An observer can verify sensory facts at this level of consciousness without necessarily implying cognition [13].

Knowledge is considered a collection of experience, appropriate information, and skilled insight that offers a structure for estimating and integrating new experiences and information. Due to the recent development of science and technology, knowledge has become an essential part of every organization. In organizations, knowledge is considered a storehouse of intelligence for the development of the organizations. One of the greatest challenges in the building of intelligent systems in every organization is the achievement of new knowledge [21].

Attitude can be defined as the way in which a person views and evaluates something or someone, a predisposition, or a tendency to respond positively or negatively toward a certain idea, object, person, or situation. It is traditionally structured along three dimensions: cognitive (perceptions and beliefs), affective (likes and dislikes, feelings, or evoked emotions), and behavioral (actions or expressed intentions toward the object based upon the “cognitive” and “affective” responses) [20].

Skills are typically defined as the mastered capacity to achieve predetermined consequences with the greatest degree of certainty, frequently with the least amount of effort, energy, or both. When referring to knowledge gained via training and experience, the term “skills” is used. These include trade and craft skills learned through apprenticeship as well as the high-level performance found in many fields, including professional practice, the arts, gaming, and athletics (Zhang, 2022).

## Significance of the study

The delivery of healthcare has been transformed by widespread Internet access and technological breakthroughs to prevent and treat the world’s major causes of mortality and disability. Mobile health technology facilitates both clinical interactions between patients and clinicians as well as non-clinical care delivery (such as health education and data reporting and monitoring). This therapeutic application, known as telemedicine, enables healthcare professionals to consult with patients remotely using a variety of modalities, including video conferencing. Telemedicine has transformed healthcare delivery in crisis situations, such as the COVID-19 pandemic [8]. Among other advantages, the use of telemedicine enhances follow-up treatment, guarantees patient access to services, and enables medical professionals to treat patients from home and in remote locations. Applications of telemedicine include decision-making, record storage and distribution, diagnostic assessment, and training of medical personnel [5]. Therefore, telemedicine is important, and it is crucial that it be included in the medical curriculum to increase acceptance and enhance the quality and safety of telemedicine practice [27]. So, this study will be used to assess awareness, knowledge, attitude, and skills of telemedicine among mental healthcare providers.

### Aim of the study

The aim of the present study was to assess awareness, knowledge, attitude, and skills of telemedicine among mental healthcare providers.

### Research questions

- Q1: What is the distribution of the studied variables (awareness, knowledge, attitude, and skills) of telemedicine among mental healthcare providers?
- Q2: Are there differences between the levels of the studied variables among mental healthcare providers (psychiatrists, nurses, and clinical psychologists)?
- Q3: Is there a relation between socio-demographic characteristics and the studied variables among healthcare providers?

## Methods

### Design

A cross-sectional design was conducted, and data were collected online from the Zagazig governorate.

### Setting

The study was conducted in three settings: psychology department lectures at the Faculty of Arts, the psychiatric outpatient clinic, and (Al-Salam building, psychiatric inpatient department) at Zagazig University and Hospitals.

- Faculty of Arts: which consists of three floors. The first floor is designated for the faculty’s employees, including staff and faculty members, and the other two floors include rooms for teaching theoretical subjects, a place where study subjects were chosen (psychology department).
- Outpatient clinic: which consists of six floors. The psychiatric outpatient clinic is enrolled on the fourth floor, which is designated for employees, including psychiatric faculty members, patients, nursing staff, and workers.
- Al-Salam Building, Sednaoui Hospital: which consists of six floors. Each floor is designated for specific departments for caring for patients, like neurology and psychiatry. The psychiatric inpatient department is enrolled on the second floor, which is designated for the employees, including psychiatric faculty members, patients, nursing staff, and workers.

### Subjects

The study samples included 264 healthcare providers, including psychiatrists, nurses, and clinical psychologists. Participants in the study had to meet the following requirements: accepted to take part in the research, ranging in age from < 30 to 60 years, and from both sexes.

### Sample technique

A purposive sampling technique was used in this study according to the following criteria: psychiatrists working in outpatient and inpatient clinics; psychologists enrolled in their fourth year at the Faculty of Arts, psychology department; and nurses working in the psychiatric department, inpatient and outpatient.

### Sample size

The population size for the 784 psychologists, 35 psychiatrists, and 25 nurses, according to Stephen Thompson’s equation, is the following:

$$n=\frac{N\times p\left(1-p\right)}{\left[\left[N-1\times \left({d}^{2}{z}^{2}\right)\right]+p\left(1-p\right)\right]}$$

*N* = population size; *z* = the standard score corresponding to the significance level of 95%, which is equal to 1.96%; *d* = error margin equal to 5%; and *p* = availability of the phenomenon in the population = 50%. By applying the equation, the total sample size is 294: 253 psychologists, 28 nurses, and 14 psychiatrists. After the pilot study, the total sample size is 264: 227 psychologists, 25 nurses, and 12 psychiatrists.

### `

During the pilot study, social networks such as WhatsApp and Facebook were the main platforms for the distribution of the questionnaire. Then, the main sample was collected by using the face-to-face technique.

#### Tool I: Socio-demographic characteristic sheet

It included questions about age, gender, profession, and scientific degree, as well as several general questions related to telemedicine experience, etc.

#### Tool II: AKAS questionnaire

It was designed by Zayapragassarazan and Kumar [28] and modified by the researchers according to culture and recent literature reviews. The tools consisted of 4 dimensions, as follows:

1. *Awareness dimension*: The dimension consisted of 10 items after modification and measured the awareness of mental healthcare providers toward telemedicine. Responses were measured on a 3-point dimension: 1 = do not know, 2 = have heard of it, and 3 = know about it.
2. *Knowledge dimension*: The dimension consisted of 8 items after modification and measured the knowledge of mental healthcare providers toward telemedicine. Responses were measured on a 2-point dimension (1 = yes, 2 = no).
3. *Attitude dimension*: The dimension consisted of 11 items after modification and measured the attitude of mental healthcare providers toward telemedicine. Responses were measured on a 5-point dimension (1 = strongly agree, 2 = agree, 3 = undecided, 4 = disagree, and 5 = strongly disagree).
4. *Skills dimension*: The dimension consisted of 12 items after modification and measured the skills of mental healthcare providers toward telemedicine. Responses were measured on a 4-point dimension (1 = unskilled, 2 = learner, 3 = average, and 4 = expert).

### Scoring system of AKAS

For each AKAS sector, the raw scores were calculated. The range for the entire sample of the AKAS was computed, along with the mean, standard deviation, and subsamples. Additionally, the raw AKAS scores were transformed into a percentage. According to the investigators’ judgment, scores between 50 and 70% were seen as ordinary with respect to AKAS, scores between 71% and above were regarded as high with respect to AKAS, and scores between equal to and above 71% were regarded as low with respect to AKAS.

### Validity and reliability of the AKAS questionnaire

Tools were translated into Arabic language utilizing the translation and back translation method to ensure their unique validity. The content validity of the instruments was evaluated by inquiring five specialists from the academic staff at the workforce of Medicine Faculty-Zagazig University (psychiatric medicine), who re-examined the instruments for clarity, significance, comprehensiveness, understanding, and ease of application. Their proposals and recommendations were taken into consideration. *Reliability*: The reliability of the instruments was evaluated by Cronbach’s alpha test in the Statistical Package for Social Science (SPSS), version 23. They showed a good level of reliability, as follows:

**Table:** 

Awareness	Knowledge	Attitude	Skills
Cronbach’s alpha = 0.784	Cronbach’s alpha = 0.625	Cronbach’s alpha = 0.720	Cronbach’s alpha = 0.890

### Pilot study

A pilot study was conducted on 10% (30) (2 psychiatrists, 3 nurses, and 25 clinical psychologists) of the recruited sample to assess the clarity and significance of the instruments, as well as to estimate the required time for filling out the tools. The researchers requested that participants fill out the questionnaire and note any questions that were vague or difficult to reply to. The required changes were done as specific rephrasing, using easier semantics for the explanations. Those who participated in the pilot study were excluded from the main study sample.

### Fieldwork

#### The preparatory phase

Once permission was granted to proceed with the study, the researcher obtained the population size for all the studied samples. Then, the researcher met with the vice deans for the students’ affairs and education in the Faculty of Arts and the head of the psychiatric medicine department and explained to them the study aims and procedures as well as the data collection forms including obtaining official permission for the approval to conduct the study and get access to the studied sample. Content validity was checked, and tools were revised by a five-panel of experts, through the distribution of the four tools with a cover letter and explanation sheet that explains the study purpose. The preparatory phase was executed in 1 month starting on the first of April and was completed by the end of the same month and year.

#### The assessment phase

The assessment phase involved actual contact with the studied sample. It was necessary to take safety precautions in response to the COVID-19 pandemic, including keeping a physical distance, donning a mask, keeping rooms well-ventilated, avoiding crowds, and washing hands. Then, the researcher met with the studied sample with a list of the names.

The researchers conducted a web-based survey to assess awareness, knowledge, attitude, and skills of telemedicine among mental healthcare providers. They received a link to the survey and answered online. Furthermore, the pilot study informed us to make face-to-face data collection and some modifications in the tools and translation into Arabic.

The researchers introduced themselves; obtained written consent from the studied sample; explained the purpose, nature, and importance of the study to the studied sample; and explained that information would be handled confidentially and would only be used for scientific research. Then, the data collection forms were explained, as researchers explained that data collection consisted of two tools: the first was socio-demographic characteristics such as age, profession, scientific degree, and certain questions about telemedicine practice. The second tool was called as the AKAS questionnaire, which is divided into four dimensions (awareness, knowledge, attitude, and skills). The studied sample was asked to read each paragraph carefully and slowly and then choose the answer by applying a mark (✓) in front of the staging that suited them. The pilot study’s results revealed that the average time to fill in all the tools was 20–35 min, 5 min for the socio-demographic characteristics sheet, and 15 min for the AKAS questionnaire. After data collection, the researcher and a specialist in statistics conducted all necessary steps for checking the completeness of the data and proceeded to the scoring of the members’ answers. The researchers collected the data first from the Faculty of Arts, followed by the outpatient clinic, and finally Al-Salam Building (psychiatric inpatient clinic). The assessment phase takes 3 months, from May to August. The fieldwork lasted 4 months, from the beginning of April 2022 to the end of August 2022.

### Statistical analysis

SPSS 23.0 for Windows was used to gather, compute, and statistically analyze the data (SPSS Inc., Chicago, IL, USA). Range and qualitative data were expressed as absolute frequencies (number) and relative frequencies, whereas quantitative data were expressed as the mean SD (percentage). The chi-square test was used to compare between levels of each dimension and between professions (psychiatrists, nurses, and clinical psychologists). A two-way ANOVA test was used in all relations of demographic with the studied dimensions.

## Results

Two hundred sixty-four healthcare providers participated in the present study. Based on Tables 1, 2, and 3, 87.9% of the studied sample were aged less than or equal to 30 years, 83% of them were female, 86% were psychologists, and 85.2% were enrolled in license as a scientific degree. 56.4% of the studied sample does not have a telemedicine unit in the hospital, 84.8% used telemedicine for remote consultants, 92.8% spent time in individual and group therapy by telemedicine, and 93.2% spent time in assessment by telemedicine. These tables also reveal that 59.8% of the studied sample used communications apps as a means of providing telemedicine. 37.1% of them reported that easy access to the processor anywhere is the benefit of telemedicine, and 39.8% of the studied sample reported that distance was a reason for using telemedicine and absence of empathy was a reason for not using telemedicine, respectively. So, these tables demonstrated that 51.1% of them did not prefer to use telemedicine for specific psychotherapies.

**Table 1:** Socio-demographic characteristics among mental healthcare providers (*n* = 264)

Socio-demographic characteristics	Items	*N*	%
Age	30 ≥	232	**87.9**
	31–40	20	7.6
	41–50	9	3.4
	51–60	3	1.1
Gender	Male	45	17.0
	Female	219	**83.0**
Profession	Psychologist	227	**86.0**
	Nurse	25	9.5
	Psychiatrist	12	4.5
Scientific degree	License	225	**85.2**
	Bachelor	6	2.3
	Diploma	22	8.3
	Master	5	1.9
	Doctorate	6	2.3
**Total**		**264**	**100.0**

**Table 2:** Telemedicine practice among mental healthcare providers (*n* = 264)

Telemedicine practice	Items	*N*	%
Having a telemedicine unit in college/hospital?	No	149	**56.4**
	Yes	115	43.6
The purpose is telemedicine used?	Remote consulting	224	**84.8**
	Continuous education	35	13.3
	I do not know	5	1.9
Percentage of time spent in individual and group therapy by telemedicine	0–25%	245	**92.8**
	26–50%	14	5.3
	51–75%	4	1.5
	76–100%	1	.4
Percentage of time spent in assessment by telemedicine	0–25%	246	**93.2**
	26–50%	13	4.9
	51–75%	5	1.9
Means of telemedicine	Communication apps	158	**59.8**
	E-mail	9	3.4
	Medical applications	15	5.7
	Smartphones	82	31.1
**Total**		264	**100**

**Table 3:** Telemedicine usage among mental healthcare providers (*n* = 264)

Telemedicine usage	Items	*N*	%
Benefits of telemedicine	Avoid stigma	39	14.8
	Reduce effort	22	8.3
	Reducing contact (COVID-19)	35	13.3
	Time-saving	60	22.7
	Reduce costs	10	3.8
	Easy access to the processor anywhere	98	**37.1**
Reasons for using telemedicine	Outback	37	14.0
	Distance	105	**39.8**
	Clinics answer stigma	43	16.3
	Emergency cases	21	8.0
	A doctor or patient travel	45	17.0
	Difficulty transporting the patient	13	4.9
Reasons for not using telemedicine	Record sessions	17	6.4
	Lack Internet	59	22.3
	Network space not secured	11	4.2
	Privacy is not guaranteed	28	10.6
	No guarantee of confidentiality	42	15.9
	Not suitable for emergencies	11	4.2
	Absence of empathy	68	**25.8**
Telemedicine usage in specific psychotherapies	No	139	51.1
	Dialectical behavior therapy	15	5.7
	Acceptance and commitment therapy	51	19.3
	Cognitive behavioral therapy	63	23.9
**Total**		**264**	**100.0**

**Fig. 1:**
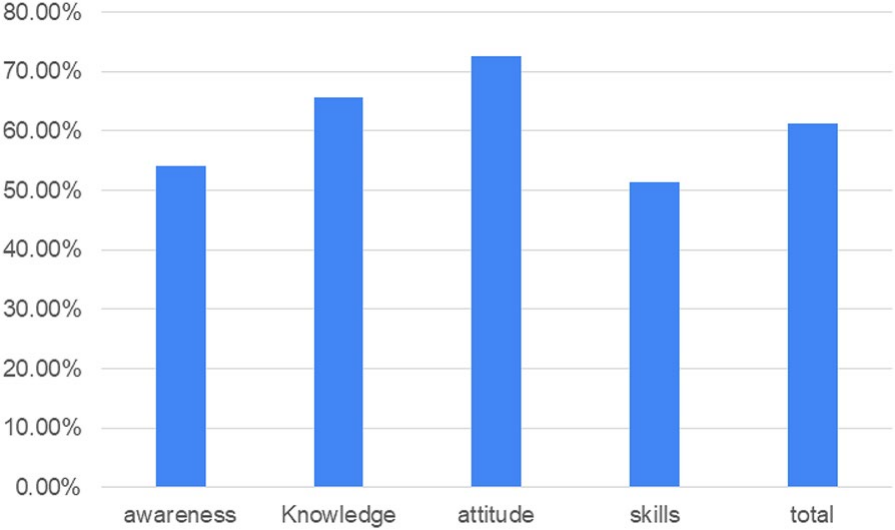
Distribution of the studied variables among the studied sample (*n* = 264)

**Fig. 2:**
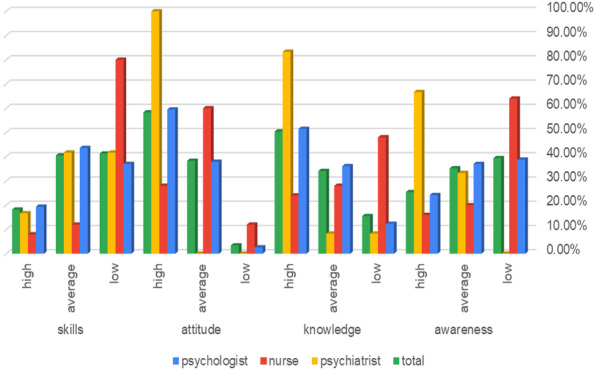
Difference of the studied variables among the studied sample (*n* = 264)

The percentage of the attitude dimension referred to a high positive level of the total sample toward telemedicine, and the percentages of awareness, knowledge, skills, and total scores referred to an average level as seen in Fig. 1. There was a high percentage among the studied psychiatrists in the studied variables (awareness, attitude, knowledge, and skills), followed by psychologists, while the nurses became the lowest in all studied variables and the studied psychologists pointed to the average level as seen in Fig. 2.

Regarding the age and gender of the studied variables, there are statistically significant differences in the dimensions of the telemedicine questionnaire according to age, where the *F* values ​were statistically significant for the level of significance (0.01). Knowledge and attitude rise in the direction of those aged less than or equal to 30 years, and the total degree and skills dimension rise in the direction of both those under or equal to 30 and over 50 years old. There are no statistically significant differences in the dimensions of the telemedicine questionnaire according to gender, where the values of *F* were not statistically significant for the level of significance (0.01) in all dimensions of the questionnaire and in its total degree, which indicates the convergence of the estimates of males and females for the dimensions of the questionnaire as seen in Table 4.

**Table 4:** Relation of the studied variables according to age and gender (*n* = 264)

	**Gender**		**Age**			
	**Male**	**Female**	** 30 ≥**	**31–40**	**41–50**	**51–60**
**Awareness:**						
***N***	45	219	232	20	9	3
**Mean**	10.38	10.95	11.12	7.20	9.56	18.33
**SD**	5.353	4.750	4.377	6.075	8.560	2.517
***F***	.518		7.063			
**Sig**	.472		.001			
**Knowledge:**						
***N***	45	219	232	20	9	3
**Mean**	5.69	5.17	5.41	4.15	4.33	3.33
**SD**	1.6	1.94	1.802	2.540	3.122	2.517
***F***	2.638		4.407			
**Sig**	.106		.005			
**Attitude:**						
***N***	45	219	232	20	9	3
**Mean**	30.58	28.74	29.32	28.10	26.11	23.67
**SD**	4.304	4.784	4.536	5.220	7.079	5.508
***F***	5.692		3.017			
**Sig**	.018		.030			
**Skills:**						
***N***	45	219	232	20	9	3
**Mean**	18.82	18.48	19.54	10.55	10.78	17.33
**SD**	7.187	8.048	6.992	9.971	8.700	16.623
***F***	.070		12.431			
**Sig**	.7		.001			
**Total**						
***N***	264					
**Mean**	18.54					
**SD**	7.896					
**Total:**						
***N***	45	219	232	20	9	3
**Mean**	65.47	63.34	65.40	50.00	50.78	62.67
**SD**	13.792	14.715	12.527	20.053	25.430	19.399
***F***	.797		10.312			
**Sig**	.373		.001			

Two-way ANOVA test

Regarding the profession and scientific degree of the studied variables, there were statistically significant differences in the dimensions of the telemedicine questionnaire according to the profession, where the *F* values were statistically significant for the level of significance (0.01). There were statistically significant differences in the dimensions of the telemedicine questionnaire according to the academic degree, where the values of *F* were statistically significant for the level of significance (0.01) and Ph.D. compared to diploma holders as seen in Table 5.

**Table 5:** Relation of the studied variables according to profession and scientific degree (*n* = 264)

	**Profession**			**Scientific degree**				
	**Psychologist**	**Nurse**	**Psychiatrist**	**License**	**Bachelor**	**Diploma**	**Master**	**Doctorate**
**Awareness:**								
***N***	227	25	12	225	6	22	5	6
**Mean**	10.96	7.12	16.50	10.91	11.83	6.95	17.20	16.67
**SD**	4.292	6.845	3.920	4.269	6.735	6.993	2.683	4.033
***F***	17.549			8.859				
**Sig**	.001			.001				
**Knowledge:**								
***N***	227	25	12	225	6	22	5	6
**Mean**	5.37	3.44	6.92	5.35	5.50	3.41	7.20	6.67
**SD**	1.798	2.364	1.621	1.79	2.665	2.404	1.304	1.966
***F***	17.297			7.753				
**Sig**	.001			.001				
**Attitude:**								
***N***	227	25	12	225	6	22	5	6
**Mean**	29.31	25.40	31.83	29.28	28.83	25.27	33.00	31.50
**SD**	4.589	5.204	2.725	4.592	5.947	4.968	3.082	2.665
***F***	10.487			5.184				
**Sig**	.001			.000				
**Skills:**								
***N***	227	25	12	225	6	22	5	6
**Mean**	19.55	8.72	19.92	19.47	16.17	7.64	23.20	22.17
**SD**	6.947	9.529	7.681	6.924	6.242	9.449	8.408	7.414
***F***	25.307			14.677				
**Sig**	.001			.001				
**Total:**								
***N***	227	25	12	225	6	22	5	6
**Mean**	65.19	44.68	75.17	65.00	62.33	43.27	80.60	77.00
**SD**	12.503	18.508	9.852	12.390	12.160	19.159	12.740	6.325
***F***	32.538			17.882				
**Sig**	.001			.001				

Two-way ANOVA test

Based on Table 6, there was a statistically significant relation between the level of awareness of telemedicine and each of the following: heard about the term “telemedicine” and the percentage of time spent in individual and group therapy by telemedicine. The results of the table show that there is a statistically significant relation between the level of awareness of telemedicine and each of the following: benefits of providing remote mental health services, reasons for using remote mental health services, causes of not using telemedicine, and telemedicine usage for specific treatments.

**Table 6:** Relation of awareness level according to telemedicine (*n* = 264)

Telemedicine	Items	Awareness level			Chi-square	Sig
		**Low**	**Average**	**High**		
Hearing about the term telemedicine	No	83	43	23	40.361	**.001**
	Yes	21	50	44		
	Yes	94	87	58		
Percentage of time spent in individual and group therapy by telemedicine	0–25%	94	91	60	13.129	**.041**
	26–50%	9	1	4		
	51–75%	1	0	3		
	76–100%	0	1	0		
Benefits of using telemedicine	Avoid stigma	15	18	6	50.990	**.001**
	Reduce effort	18	4	0		
	Reducing contact (COVID-19)	11	13	11		
	Time-saving	35	12	13		
	Reduce costs	3	7	0		
	Easy access to the processor anywhere	22	39	37		
Causes of using telemedicine	Outback	5	19	13	35.693	**.001**
	Distance	49	27	29		
	Clinics answer stigma	12	18	13		
	Emergency cases	6	12	3		
	A doctor or patient travel	29	10	6		
	Difficulty transporting patient	3	7	3		
Causes of not using telemedicine	Record sessions	13	8	7	38.349	**.001**
	Lack Internet	3	10	4		
	Network space not secured	32	17	10		
	Privacy is not guaranteed	2	1	8		
	No guarantee of confidentiality	9	15	4		
	Not suitable for emergencies	21	14	7		
	Absence of empathy	3	2	6		
Telemedicine usage for specific treatments	No	63	42	30	17.070	**.009**
	DBT	9	5	1		
	ACT	19	16	16		
	CBT	13	30	20		

Chi-square test

A statistically significant relation was found between the level of knowledge of telemedicine and each of the following: hearing about the term “telemedicine,” percentage of time spent in individual and group therapy by telemedicine, means of providing telemedicine, benefits of providing telemedicine, reasons for using telemedicine, causes of not using telemedicine, and telemedicine usage for specific psychotherapies as seen in Table 7.

**Table 7:** Relation of knowledge level according to telemedicine (*n* = 264)

Telemedicine	Items	Knowledge level			Chi-square	Sig
		**Low**	**Average**	**High**		
Hearing about the term telemedicine	No	17	55	57	29.836	**.001**
	Yes	4	35	76		
Percentage of time spent in individual and group therapy by telemedicine	0–25%	33	86	126	17.023	**.009**
	26–50%	7	4	3		
	51–75%	12	0	3		
	76–100%	0	0	1		
Means of providing telemedicine	Communication apps	19	53	86	18.076	**.021**
	E-mail	0	3	6		
	Medical applications	7	4	4		
	Smartphones	15	30	37		
Benefits of providing telemedicine	Avoid stigma	14	12	13	34.70	**.001**
	Reduce effort	1	7	14		
	Reducing contact (COVID-19)	0	12	23		
	Time-saving	15	25	20		
	Reduce costs	2	3	5		
	Easy access to the processor anywhere	9	31	58		
Reasons for using telemedicine	Outback	3	12	22	23.40	**.001**
	Distance	23	27	55		
	Clinics answer stigma	9	13	21		
	Emergency cases	0	12	9		
	Doctor or patient travel	2	21	22		
	Difficulty transporting the patient	4	5	4		
Causes of not using telemedicine	Record sessions	4	8	16	28.58	**.012**
	Lack Internet	2	5	10		
	Network space not secure	14	14	31		
	Privacy is not guaranteed	0	3	8		
	No guarantee of confidentiality	8	12	8		
	Not suitable for emergencies	5	24	13		
	Absence of empathy	2	3	6		
	Record sessions	6	21	41		
Telemedicine usage for specific psychotherapies	No	63	42	30	17.070	**.009**
	DBT	9	5	1		
	ACT	19	16	16		
	CBT	13	30	20		

Chi-square test

Also, a statistically significant relation was found between the level of attitude towards telemedicine and each of the following: hearing about the term “telemedicine,” willingness to attend any telemedicine training program, percentage of time spent in individual and group therapy by telemedicine, percentage of time spent in the telemedicine assessment, benefits of telemedicine, reasons for using telemedicine, and telemedicine usage in specific psychotherapies as seen in Table 8.

**Table 8:** Relation of attitude level according to telemedicine (*n* = 264)

Telemedicine	Items	Attitude level			Chi-square	Sig
		**Low**	**Average**	**High**		
Hearing the term telemedicine	No	7	70	72	14.346	**.001**
	Yes	2	31	82		
Willing to attend any telemedicine training program	No	5	11	9	24.896	**.001**
	Yes	4	90	145		
Percentage of time spent in individual and group therapy by telemedicine	0–25%	96	103	46	13.112	**.041**
	26–50%	11	3	0		
	51–75%	1	1	2		
	76–100%	1	0	0		
Percentage of time spent in the telemedicine assessment	0–25%	97	103	46	9.521	**.049**
	26–50%	10	3	0		
	51–75%	2	1	2		
Benefits of telemedicine	Avoid stigma	0	22	17	28.023	**.002**
	Reduce effort	0	4	18		
	Reducing contact (COVID-19)	2	18	15		
	Time-saving	2	26	32		
	Reduce costs	2	3	5		
	Easy access to the processor anywhere	3	28	67		
Reasons for using telemedicine	Outback	14	15	8	40.59	**.001**
	Distance	24	55	26		
	Clinics answer stigma	29	10	4		
	Emergency cases	9	7	5		
	A doctor or patient travel	29	12	4		
	Difficulty transporting the patient	4	8	1		
Telemedicine usage in specific psychotherapies	No	7	58	70	16.549	**.011**
	DBT	0	10	5		
	ACT	2	17	32		
	CBT	0	16	47		

Chi-square test

## Discussion

### Relating to socio-demographic characteristics and telemedicine practice

In the current study, more than half of the sample had no hospital-based telemedicine unit, and more than three-quarters used telemedicine for distant consulting. This may be because telemedicine was not well-known before the COVID-19 outbreak, but things have changed since then. Telemedicine technology is now a way to provide patients with quality treatment without raising the danger of COVID-19 transmission to patients during in-hospital clinic visits. Additionally, telemedicine helps medical professionals since it lowers the risk of contracting infections, eases hospital stress, gives them an appropriate instrument for treating and monitoring patients, and offers a good method for offering mental health treatments. In the same line, Carrillo et al. (2022), who studied the effectiveness of teleconsultations in primary care, reported that teleconsultations over the phone or through videoconference are a useful alternative to face-to-face consultations for many individuals utilizing primary care and mental health services. On the contrary, Abdel Nasser et al. [1], who studied measuring the patients’ satisfaction about telemedicine used in Saudi Arabia during the COVID-19 pandemic, found that old age participants in our study would prefer to use face-to-face consultation in the future rather than telemedicine,this may be due to the older generation’s need for in-person contact to discuss more emotional issues and to express all the concerns verbally and nonverbally.

In the present study, the vast majority of respondents spent time in individual therapy, group therapy, and assessment by telemedicine. This might be owing to the importance of individual and group therapy inpatient treatment, as it allows the patient to build a trusting relationship with a counselor that will not judge them, discuss feelings and problems, thus freeing themselves from burdens, and allows the counselor to teach a variety of important coping skills that help the individual manage the stress in their lives. In the same line, Sugarman et al. [25], who studied patients’ perceptions of telehealth services for outpatient treatment of substance use disorders during the COVID‐19 pandemic, reported that most respondents getting outpatient substance use disorder care found telehealth sessions to be an acceptable treatment modality, especially those receiving individual therapy. Ratings were in the majority for individual therapy and more than half for group therapy. Challenges remain for telemedicine group therapy.

In the present study, more than one-third of the studied sample reported that having easy access to a psychiatrist anywhere is the benefit of telemedicine. This may be owing to the abstention of many people from going to a psychiatrist in some cases (such as avoiding the stigma of going to psychiatric clinics, or being in remote areas that make it difficult to reach a therapist faster and more conveniently), as telemedicine provides better access. Because not everyone who has an ongoing relationship with a doctor can go to them when they need it, many online medical networks provide access at any time of the day and night or anywhere. In the same line, Arumugam et al. [6], who studied the perception of doctors regarding telemedicine use for general practice in Chennai, reported that nearly two-thirds of physicians say that healthcare services can be easily accessed through the practice of telemedicine, and nearly three-fifths of physicians would like to continue telemedicine practice even after the COVID-19 lockdown period.

In the present study, more than two-fifths of the studied sample reported distance as the reason for using telemedicine. This may be owing to eliminating the travel barrier for those living in rural or isolated communities, as well as for patients who may have limited mobility, increasing accessibility to care. This increased accessibility provides the opportunity for patients to have a more involved role in their health by reducing the distance barrier. In the same line, Ross et al. [23], who studied the implementation and patient experience of outpatient teleneurology, reported that virtual visits reduced travel significantly and increased patient satisfaction. The significant percentage of nearby patients would suggest that telemedicine offers significant access for reasons other than proximity to patients.

In the present study, more than one-fifth of the studied sample reported the absence of empathy as a reason for not using telemedicine. This may be owing to the gap in communication between psychiatrist and patient, as well as the loss of non-verbal cues and touch. On the contrary Cheshire et al. [11], who studied the patient perception of physician empathy in stroke telemedicine, found that there was no difference in how patients perceive doctors’ empathy during acute stroke therapy between in-person and telemedicine consultations. This could be because, in a telemedicine encounter, empathy can be conveyed through voice, facial expression, and attentiveness and, in the context of acute stroke treatment, does not require physical touch or closeness.

In the present study, half of the studied sample did not prefer to use telemedicine for specific psychotherapies. This may be owing to the inability to see and hear all the verbal and non-verbal communication of the patient. Also, it is difficult for them to respond quickly and effectively in crisis situations or serious psychiatric illnesses.

### Differences between the levels of the studied variables among mental healthcare providers (psychiatrists, nurses, and clinical psychologists)

In the present study, psychiatrists had the highest awareness of telemedicine. This might be because physicians are engaged in more courses, workshops, and conferences than other healthcare workers, and this allows them to be more up-to-date with the latest technologies. In the same line, Shouman et al. [24], who studied the awareness and attitude of healthcare workers towards telehealth in Cairo, Egypt, found that telehealth awareness was the highest among physicians than other healthcare workers, as it represented about half of the total awareness.

In the present study, psychiatrists had the highest knowledge of telemedicine. This might be owing to their continuing knowledge of the latest developments in healthcare providing. In the same line, Alghamdi et al. [4], who studied telepsychiatry: knowledge, effectiveness, and willingness, assessments of psychiatrists in Saudi Arabia, found that psychiatrists’ knowledge regarding telepsychiatry was deemed insufficient; more than half of the psychiatrists showed a poor level of knowledge regarding telepsychiatry.

The studied psychiatrists had the highest attitude toward telemedicine. This may be owing to the psychiatrists’ being in contact with patients and familiar with the various and advanced psychological treatments associated with dealing with patients, such as telemedicine and telepsychiatry, especially after the COVID-19 pandemic. On the contrary, Biruk et al. (2018), who studied the knowledge and attitude of health professionals toward telemedicine in resource-limited settings in northwest Ethiopia, found that nurses had a good attitude toward telemedicine.

In the present study, skills had an average mean among the studied sample. This might be owing to the unavailability of computer and Internet services for some of the studied samples. In the same line, Elhadi et al. [14], who studied telemedicine awareness, knowledge, attitude, and skills of healthcare workers in a low-resource country during the COVID-19 pandemic in Libya, found that only one-third of the studied sample had computer skills when compared to other variables; knowledge and attitude were more than three-quarters, and awareness was more than half.

### Concerning awareness relation with socio-demographic characteristics

In the present study, awareness was average among the studied sample. This might be owing to the limited talk about telemedicine and its importance, as well as the limited training and awareness courses and programs that are offered about telemedicine. This might also be because the majority of the sample were psychologists, and they did not have enough experience in the field of work or in the ways of providing mental healthcare services through telemedicine. Another study conducted in India [12] reported that the majority of participants had high scores for awareness, knowledge, and attitude toward telemedicine.

In the present study, the studied sample whose age group was between 41 and 50 years had the highest level of awareness. This might be owing to their experience in providing medical care. On the contrary, Zayapragassarazan and Kumar [28], who studied awareness, knowledge, attitude, and skills of telemedicine among health professional faculty working in teaching hospitals, found that the highest mean value for awareness of telemedicine was recorded among the respondents whose age group was between 30 and 40 years.

In the present study, females had the highest awareness. This might be owing to the desire of females more than males to develop themselves; the continuous interest in attending training courses, workshops, and conferences; and the aspiration to know everything new and updated in the world of providing medical services, especially during the period of the COVID-19 pandemic. In the same line, Elhadi et al. [14], who studied telemedicine awareness, knowledge, attitude, and skills of healthcare workers in a low-resource country during the COVID-19 pandemic, reported that females had significantly higher mean awareness than males.

In the present study, the master’s degree holders of the studied sample had the highest awareness regarding telemedicine. This might be owing to their continuous knowledge of the latest means of providing medical care, as well as the ability to adapt to modern and emerging scientific developments thanks to their experiences. In the same line, Shouman et al. [24], who studied the awareness and attitude of healthcare workers towards telehealth in Cairo, Egypt, found that two-thirds of healthcare workers with master’s degrees were aware of telehealth, compared to around one-third of those with a diploma.

### Regarding knowledge relation with socio-demographic characteristics

In the present study, knowledge was average among the studied sample. This might be because there was no urgent need to use it before, it was not highlighted enough. This may change because the current epidemiological situation in the country has revealed the importance of telemedicine, which has given impetus to a greater number of telemedicine adoptions. In the same line, Albarrak et al. [3], who studied the assessment of physicians’ knowledge, perception, and willingness of telemedicine in Riyadh region, Saudi Arabia, reported that more than two-fifths of participants have average knowledge about telemedicine technology.

In the present study, the studied sample, who had less than or equal to 30 years of age, had the highest knowledge. This might be owing to being young and enthusiastic to learn about the latest developments and technology that serve the field of work, and because there was no urgent need to use it before that, it was not highlighted enough. This may change because the current epidemiological situation in the country has revealed the importance of telemedicine, which has given impetus to a greater number of telemedicine adoptions. In the same line, Ahmed et al. [2], who studied knowledge, attitudes, and perceptions related to telemedicine among young doctors and nursing staff at the King Abdul-Aziz University Hospital Jeddah, KSA, found that when compared to the senior staff participants in the study, the youthful nursing staff’s knowledge and attitude were relatively better.

In the present study, females had the highest knowledge. This might be because females tend to seek more information related to health issues by attending conferences, speeches, or meetings held regarding telemedicine technology. In the same line, Umayam et al. [26], who studied knowledge, attitudes, and perceptions on the use of telemedicine among adults aged 18–34 in Manila, Philippines, during the COVID-19 pandemic, found that females had a higher mean knowledge score compared to males.

In the present study, the master’s degree holders in the studied sample had the highest knowledge regarding telemedicine. This might be because the majority of master’s degree holders are doctors and doctors are keener than others to attend conferences and workshops and aspire to develop themselves in the field of providing medical services. On the contrary, Biruk et al. (2018), who studied knowledge and attitude of health professionals toward telemedicine in resource-limited settings in northwest Ethiopia, found that more than three-quarters of health professionals who were considered to have good knowledge of telemedicine were bachelor’s degree holders.

### Concerning attitude relation with socio-demographic characteristics

In the present study, the attitude had the highest mean among the studied sample. This might be owing to the widespread use of telemedicine in medical education. It aids in providing healthcare facilities through the use of mobile health clinics and online consultations with specialists for a disease’s diagnosis, screening, and management (including follow-up). On the contrary, Malhotra et al. [18], who studied knowledge, perception, and attitude of using telehealth services among college-going students of Uttarakhand, India, found that there is a lack of familiarity and awareness of telemedicine among students.

In the present study, the studied sample, which was less than or equal to 30 years old, had the highest attitude. This might be because young people use smart boards (iPads), messaging, and software and take pictures, so they know more about technology and can deal with it proficiently. In the same line, Biruk et al. (2018), who studied the knowledge and attitude of health professionals toward telemedicine in resource-limited settings in northwest Ethiopia, found that more than half of the age group between 20 and 29 years had a good attitude toward telemedicine.

In the present study, males had the highest attitude toward telemedicine. This might be because the COVID-19 pandemic has forced some professions to work from home, and the use of Internet services has increased to maintain the continuity of work. Telemedicine was one of the applications that people used more at that time. The attitude toward telemedicine itself, the patient-physician interaction, and the level of technology usage may all have an impact on how willing a person is to utilize telemedicine. On the contrary, Elhadi et al. [14], who studied telemedicine awareness, knowledge, attitude, and skills of healthcare workers in a low-resource country during the COVID-19 pandemic in Libya, found that there was no difference in the attitude between males and females.

The master’s degree had the highest attitude toward telemedicine. This might be because telemedicine has become an important means for both the doctor and the patient as it reduces time, the patient can express his feelings, and develop the ability to talk with others, so doctors tend to use it. In the same line, Elsaie et al. [15], who studied Egyptian dermatologists’ attitude toward telemedicine amidst the COVID-19 pandemic in Egypt, found that the master’s degree holders showed the most significant attitude toward telemedicine.

### Concerning skills relation with socio-demographic characteristics

In the present study, males had the highest skills. This might be owing to secure income for family members, men’s interest in using modern advanced means such as telemedicine has increased, as they are responsible for the family and its well-being, which helps them to live a better and safe life. On the contrary, Elhadi et al. [14], who studied telemedicine awareness, knowledge, attitude, and skills of healthcare workers in a low-resource country during the COVID-19 pandemic in Libya, found that more than three-quarters of females had high computer skills, which were higher than those of males.

## Conclusions

This study concluded that the studied mental healthcare providers had high attitudes while having average among the other studied variables (awareness, knowledge, and skills). There was a high percentage among the studied psychiatrists in the studied variables (awareness, attitude, knowledge, and skills) followed by psychologists, while nurses became the lowest in all studied variables level.

## Recommendation

- Workshops, conferences, and training sessions are required to close the telemedicine knowledge gap.
- Adding telemedicine units to the mental health department in hospitals and specialized departments in colleges.
- Applying telemedicine as part of the medical curricula in colleges.
- Future research is required to ascertain how telepsychiatric treatment affects patients’ health outcomes as well as how much it costs patients and the healthcare system.

## Data Availability

The dataset generated and/or analyzed during the current study is available from the corresponding author upon reasonable request.

## Abbreviations

DBT: Dialectical behavior therapy
ACT: Acceptance and commitment therapy
CBT: Cognitive behavioral therapy
AKAS: Awareness, knowledge, attitude, and skills
